# High-Amplitude Gastric Contractions following Laparoscopic Sleeve Gastrectomy

**DOI:** 10.1155/2019/7457361

**Published:** 2019-01-20

**Authors:** Jerry Zhou, Carolyn Jameson, Vincent Ho

**Affiliations:** ^1^School of Medicine, Western Sydney University, Sydney, Australia; ^2^Sydney Bariatric Clinic, Penrith, Australia; ^3^Gastroenterology, Campbelltown Hospital, Campbelltown, Australia

## Abstract

A proportion of laparoscopic sleeve gastrectomy patients experience symptoms of regurgitation and epigastric pain postoperation. The appearance of gastric sleeve contractions has been documented but its implications have not been adequately investigated. This case describes a 61-year-old female following laparoscopic sleeve gastrectomy. The patient underwent high-resolution impedance esophageal manometry that identified compartmentalized pressurization leading to propagating contractions throughout the gastric sleeve. Combined treatment with calcium channel blockers and gastric sleeve dilation relieved all symptoms. This case highlights the application of high-resolution impedance esophageal manometry to assess motor function and bolus transit in patients following laparoscopic sleeve gastrectomy.

## 1. Introduction

Laparoscopic sleeve gastrectomy (LSG) is an increasingly utilized bariatric surgical procedure. LSG involves removal of approximately 75% of the stomach, leaving a long narrow sleeve stomach, similar in diameter to the esophagus. Following LSG, chronic complications including gastroesophageal reflux disease, nausea, epigastric pain, and regurgitation during consumption of solids and liquids are reported in 29%-46% of patients [1, 2]. These complications are incompletely understood and may be difficult to treat. Causes of post-SG symptoms have been attributed to the following: increased intraluminal gastric sleeve pressure [3, 4], narrowing or stenosis of the gastric sleeve [5], abnormal lower esophageal sphincter (LES) pressure [6, 7], and hypertensive esophagus [8]. Another potential cause of symptoms is propagating contractions from the lower esophageal sphincter into the gastric sleeve. Aside from this feature being observed in post-LSG fluoroscopy swallow studies [9, 10], little is documented on the effects of abnormal sleeve contractions on symptoms and bolus transit. We present a case of post-LSG gastric sleeve contractions demonstrated by high-resolution impedance manometry (HRiM).

## 2. Case

A 61-year-old patient with BMI 42.5 kg/m^2^ underwent an uncomplicated LSG using a 36-Fr bougie with routine dissection to 2 cm proximal to the pylorus. Other than a small hiatus hernia, preoperative barium swallow was unremarkable. The patient denied symptoms of dysphagia or gastroesophageal reflux preoperatively. Two weeks following LSG, she developed nausea, epigastric pain, and regurgitation with all liquids and solids. Postoperative barium swallow study observed free passage of contrast thorough the distal esophagus but a holdup at the esophagogastric junction (EGJ). There was unhindered passage of contrast through the gastric sleeve with minimal gastroesophageal reflux detected during the study.

Gastroscopy, performed 3 months later, observed a hiatus hernia at the EGJ and trachealisation of the esophagus. Investigation of the gastric sleeve identified mild inflammation with raised areas with intestinal metaplasia in the antrum. Histopathology from esophagus biopsies was negative for eosinophilic esophagitis, whilst gastric sleeve biopsies showed atrophic gastritis. The distal esophagus was dilated to 20 mm empirically using a Savary-Gilliard (Wilson-Cook, USA) thermoplastic dilator. Symptoms remained unchanged after distal esophagus dilation.

**Figure 1:**
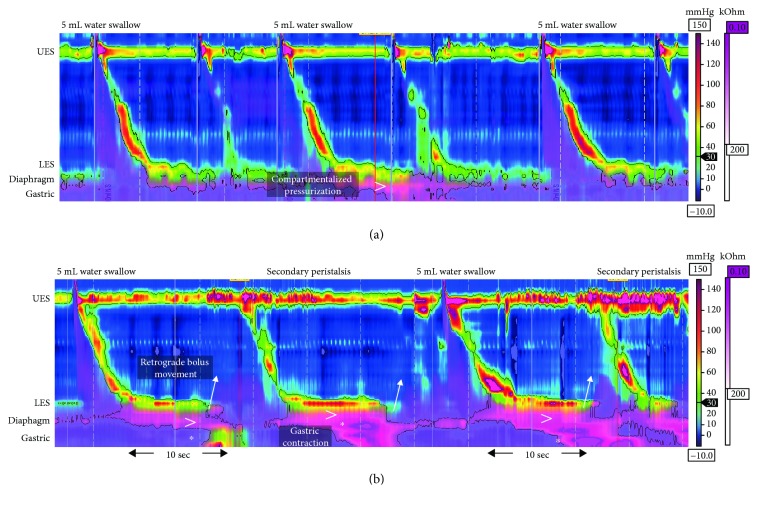
Examples of water swallows in post-LSG cases obtained with high-resolution impedance manometry at 30 mmHg isobaric contour. Impedance data are displayed by overlay pink colorization with the pink shading indicative of areas on the topography plots with bolus present. (a) An example of asymptomatic post-LSG peristaltic morphology. The 5 mL water swallows show intact peristalsis and complete bolus transit. A small amount of compartmentalized pressurization occurs at the hiatus hernia and propagates into the gastric sleeve (denoted by >). (b) Swallow morphology of the case study showing normal peristalsis until at the contractile deceleration point where compartmentalized pressurization generates high-amplitude, long-duration contraction below the diaphragm (denoted by ∗). This contraction propagates further down into the gastric sleeve. The increase in intragastric pressure correlates with transient LES relaxation which results in the retrograde movement of gastric bolus (arrows highlight incidences of this retrograde movement). The presence of bolus in the esophagus triggers an involuntary secondary peristalsis to clear the distal esophagus. This process is repeated in the second 5 mL water swallow. UES: upper esophageal sphincter; LES: lower esophageal sphincter.

**Figure 2:**
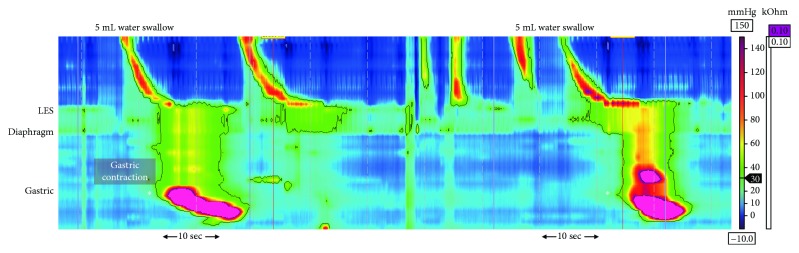
Examples of water swallows and subsequent gastric sleeve contractions obtained with high-resolution impedance manometry. The catheter was intubated 20 cm beyond the suggested position of 43 cm, to capture compartmentalized pressurization in the proximal gastric sleeve leading to propagating contractions in the distal gastric sleeve (denoted by ∗). UES: upper esophageal sphincter; LES: lower esophageal sphincter.

HRiM and 24-hour pH monitoring were performed 1 month after gastroscopy. HRiM identified a 2.6 cm hiatus hernia at the EGJ which became intensely pressurized after each test swallow. In 11 of 20 test swallows, compartmentalized pressurization was generated below the lower esophageal sphincter (LES) and propagated into the gastric sleeve, where a high-amplitude contraction generated “inverted” pressurization (Figure 1(b)). Transient LES relaxation occurred to relieve build-up of pressure which facilitated retrograde movement of the bolus. This bolus triggered a secondary peristaltic wave to clear the distal esophagus. The patient experienced epigastric pain and sensation of “food being stuck” during these gastric contractions. The HRiM catheter was intubated a further 20 cm below the LES into the gastric sleeve and additional 8 test swallows conducted. In 4 of the 8 swallows, compartmentalized pressurization at the EGJ propagated into the gastric sleeve leading to high-amplitude contractions (Figure 2). The 24-hour pH monitoring study was negative for acid reflux (DeMeester score 1.7) with a negative symptom index score (0.0%) between reflux episodes and regurgitation. Patient was prescribed calcium channel blocker (30 mg diltiazem orally three times a day) which improved symptoms; she was able to tolerate 1 L of water and 2 caffeinated drinks daily.

Five months after dilation and treatment with diltiazem, the patient remains symptomatic with solids only. Repeat gastroscopy demonstrated no clear narrowing of the esophagus or gastric sleeve. Both were empirically dilated to 20 mm.

Thirteen months following LSG and four months after the second dilation, the patient reported no further nausea, epigastric pain, or regurgitation symptoms. The patient ceased all medications including diltiazem. Her BMI was 31 kg/m^2^ which was maintained through a program of regular exercise and dietary modification.

## 3. Discussion

This case demonstrates abnormal post-LSG gastric sleeve pressurization and contraction. Previously, fluoroscopy swallow studies have suspected high-amplitude gastric sleeve contraction when patterns of contrast passage through the gastric sleeve produced a filiform or “corkscrew” appearance [9, 10]. Although no correlation was found between gastric sleeve appearance and time for contrast passage in those studies, it was unclear whether gastric sleeve distortion was maintained between swallows or occurred transiently. In our patient, the gastric sleeve compartment contracted only following bolus swallows. Bolus arriving at the EGJ generated compartmentalized pressurization at the hiatus hernia which resulted in a series of propagating contractions into the gastric sleeve. Because the gastric sleeve was not distensible, it behaved like an esophageal extension. Any bolus (liquid or solid) which traversed the LES, as a consequence of the inverted pressures generated in the gastric sleeve, underwent retrograde regurgitation through the distal esophagus.

A similar study found frequent retrograde bolus movement in post-LSG patients by HRiM [11]. Their observations were attributed to higher intraluminal pressures in the gastric sleeve. In a native stomach, the bolus reaching the gastric lumen causes distention of the fundic wall which allows intragastric pressure to be maintained. Removal of the gastric fundus during LSG causes reduction in gastric compliance and a subsequent increase in intraluminal pressure [3, 4]. As a bolus impacts against the elevated gastric pressure and it can be “reflected back” into the distal esophagus, impedance profiles of retrograde bolus movement are similar between cases of elevated intragastric pressure and gastric sleeve contractions seen here. However, manometric pressure topographies for these two abnormalities are different. The former presents as a band of pangastric pressurization that starts at the LES and extends into the gastric sleeve, whilst gastric sleeve contractions present as focal areas of high-amplitude pressure.

HRiM assessment of our patient identified features of an underlying esophageal motility disorder similar to hypertensive esophagus, albeit localized below the EGJ. These contractions below the hiatus hernia traversed the proximal gastric sleeve which caused focal areas of high contractile vigor. It is unclear whether this was present prior to LSG surgery, a preexisting condition exacerbated by LSG, or whether the condition was created by LSG. The literature suggests that LSG weakens the contraction amplitude of the LES, which can contribute to postoperative reflux [6, 7]. Another recent study used high-resolution electrical mapping to identify ectopic pacemaking and dysrhythmia in the gastric sleeves following LSG [12]. They found that a patient with nausea and vomiting after LSG had unifocal ectopic pacemaker in the distal stomach that generated rapid retrograde-propagating wavefronts. These post-LSG changes in the omnipresent gastric slow-wave pacemaking may promote abnormal gastric contractions seen in our case study.

Diltiazem, a calcium channel blocker, is a proven treatment of excessive contraction in the esophageal and associated symptoms [13]. After a combination of dilation of the sleeve, to prevent possible sleeve twisting, and prescription of diltiazem, symptoms in our patient completely resolved. In a similar report of “sleeve dysmotility syndrome,” a patient developed hypertensive esophagus post-SG and experienced ongoing dysphagia. Progression to a Roux-en-Y gastric bypass only partially relieved the symptoms [8]. Gastric bypass would have been an option if the symptoms in our patient persisted.

In this case study, the SG starting point of the antral resection was 2 cm from the pylorus, shorter than the conservative resections at 6 cm. Conservative resections have the potential of improving gastric emptying and decreasing intraluminal pressure. However, studies comparing 2 cm vs. 6 cm have not found significant increases in post-SG dysmotility or reflux [14]; specific studies looking at post-SG intragastric pressures have found no differences between the techniques [15, 16].

Fluoroscopic swallow has been the conventional tool used to assess complications following LSG. Fluoroscopy, however, requires radiation exposure and provides inadequate detail to define and classify motor function. HRiM was devised to circumvent radiation exposure in assessing bolus transit patterns whilst providing information regarding motor function. Although designed to assess physiology of the esophagus, we have demonstrated the capabilities of HRiM in both identifying post-LSG dysmotility and in guiding treatment. Abnormalities associated with gastric sleeve function and bolus transit in patients with symptoms following LSG can be assessed using this novel application of HRiM.
